# Supplementary material for: Microbial growth in actual martian regolith in the form of Mars meteorite EETA79001
Source: Commun Earth Environ. 2023 Oct 17;4(1):381. doi: 10.1038/s43247-023-01042-7 (PMC11041791; doi:10.1038/s43247-023-01042-7)
Supplement: Supplementary file 2 — Supplementary Information [file 43247_2023_1042_MOESM2_ESM.pdf]

Supplementary Information

Microbial growth in actual martian regolith in the form of Mars meteorite EETA79001

Neveda Naz, Bijan F. Harandi[†], Jacob Newmark[†], and Samuel P. Kounaves*
Department of Chemistry, Tufts University, Medford, MA 02155, USA.

[*] Correspondence: samuel.kounaves@tufts.edu
[†] Have contributed equally to this work.

Supplementary Notes 1

Bacterial and Cyanobacterial Selection Rationale

The cyanobacterium *Eucapsis* was chosen as the control for our tests due to its ease of cultivation and rapid growth. *Eucapsis* is a freshwater cyanobacterium, with a pH range of 5.5-6.5, it belongs to the *Synechococcales* order [1]. *Eucapsis* species have been found in various environments, including acidic and cold swamps, and they are also present in tropical, temperate, and sub-polar climates [2]. *Eucapsis* thrives in temperatures around 20-22°C.

Chr20 was selected as an especially unique candidate for this study. It was isolated from a quartz rock collected in the Atacama Desert, one of the most extreme and hyperarid environments on Earth. The location where the rock was collected, Yungay Salar, receives less than 20 mm of precipitation annually and has experienced decades without any rainfall [3]. It is characterized by a high UV index (11-20), scarce nutrient availability, low relative humidity (<30%), minimal soil water content (<1%), and high salt concentrations [4-8]. Given its origins in the hyperarid Atacama Desert, *Chr20* is a strong point of comparison with *Eucapsis,* especially in this study where the ratio of regolith:water was tested as an experimental parameter.

E. coli was chosen as a model aerobic organism due to its fast reproduction, and ease of maintenance [9]. It can grow at low temperatures (5.5°C to 10°C) [10,11] with a maximum temperature of around 47°C [12], and prefers an optimal pH range of 5.6-6.5 [13]. *E. coli* can grow at very low dissolved oxygen levels, as found in Martian brines, through aerobic respiration [14]. Moreover, *E. coli* strains have been recovered from spacecraft and spacecraft clean rooms, which have been used in astrobiological studies, raising concerns about planetary protection and the inadvertent introduction of terrestrial life to other worlds [15-18].

P. halocryophilus is an aerobic, gram-positive bacterium isolated from permafrost soil in the Canadian High Arctic, making it an ideal organism for studying high salt and subzero temperature activity. It can grow in environments with 19% NaCl and a pH range of 6.0-11.0, with an optimal pH of 7.5. It can tolerate temperatures ranging from -15°C to 37°C, and even shows low levels of metabolic activity in frozen permafrost microcosms down to -25°C [19-21]. *P. halocryophilus* displays viability in briny solutions due to the combination of low pressure and high salt, which provides a stable liquid medium. It is classified as an extremophile capable of both halophilic and psychrophilic growth, thriving in environments with high salinity and extremely low temperatures. It exhibits an interesting ability to increase its growth rate when exposed to various chemical solutions at temperatures ranging from -30°C to 25°C, especially in chloride solutions [22]. Previous research has indicated that higher salt concentrations in the solution further enhance the growth and survival rates of *P. halocryophilus* under martian conditions [22]. Consequently, *P. halocryophilus,* with its halophilic and psychrophilic characteristics, is an ideal candidate for studies in martian regolith.

Supplementary Methods 1

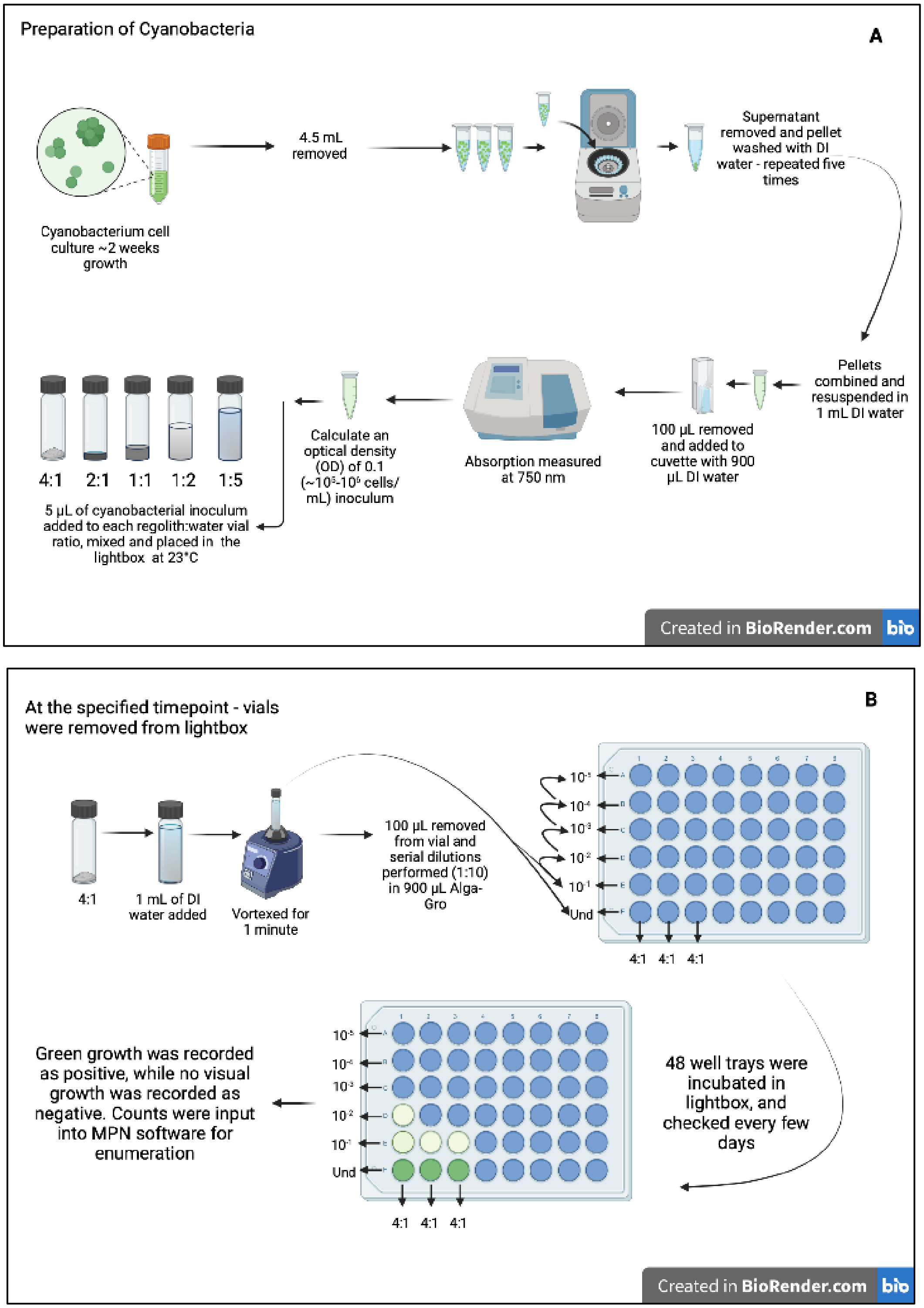

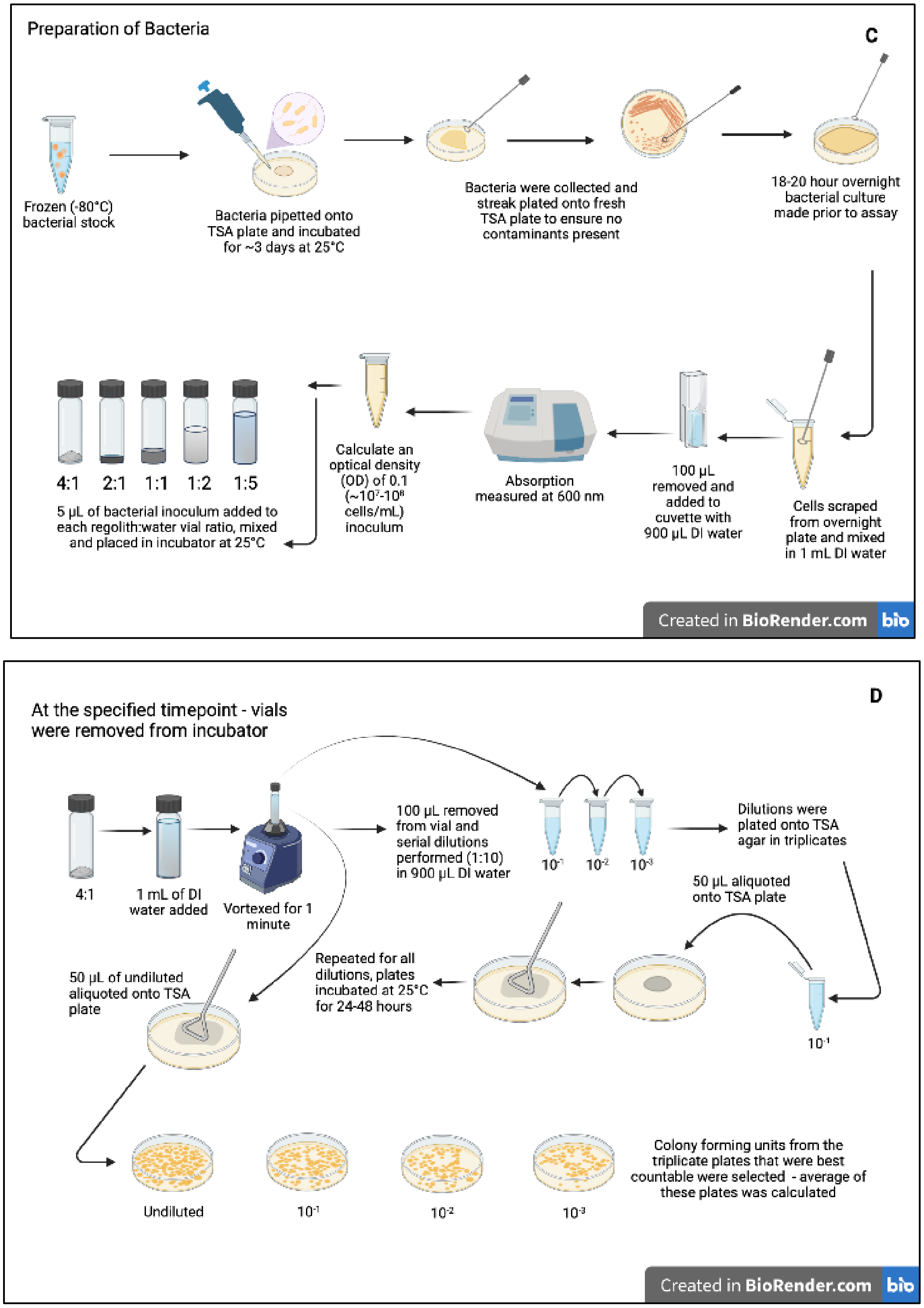

Fig. S1. Graphics Outlining Methods of Enumeration for Bacteria and Cyanobacteria. Visualization of (**A**) cyanobacteria preparation and cultivation, (**B**) MPN calculations for cyanobacteria, (**C**) bacteria preparation and cultivation, (**D**) CFU calculations for bacteria as outlined in the Methods section. *Adapted from "Microbiology Template", by BioRender.com (2023). Retrieved from https://app.biorender.com/biorender-templates*

Table S1. Ionic composition of EETA79001 sawdust, Mars regolith, and Mars simulants. Anion and cation compositions of the various samples for a regolith:water ratio of 1:5. The leaching procedure has been previously described in detail [23]. LOD: Limits of Detection. ND: Not Determined.

Ions in Leachate	EETA79001 (ppm)*	Mars-WCL (ppm)*	MMS (ppm)*	MGS (ppm)*	JSC (ppm)*
Ca^{2+}	23.7	120	10.2	960	26
Mg^{2+}	16.6	401	1.88	520	4.4
K^{+}	0.36	76.2	8.2	12.8	13.4
Na^{+}	12.7	161	48	42	17
Cl^{-}	2.3	83.3	1.5	12	2.2
NO_3^{-}	15.0	310	0.7	58	1.52
$SO_4^{=}$	56.4	2594	2.8	4200	7.2
ClO_4^{-}	0.61	1193	<LOD	< LOD	< LOD
PO_4^{3-}	7.6	ND	< LOD	< LOD	0.068
Cond. µS cm^{-1}	420	8200	248	6.4	202
pH	8.4	7.7	7.9	7.3	7.9

* Original concentrations recalculated for 1:5 ratio.

EETA79001 = Mars meteorite [23]
Mars-WCL = Phoenix Mars Lander Wet Chemistry Laboratory [23]
MMS = Mars Mojavi Simulant [24]
MGS = Mars Global Simulant [24]
JSC = NASA-JSC Mars-1 Simulant [24]

Supplementary References

1 Komárek, J., Hindák, F. & Jezberova, J. Review of the cyanobacterial genus Eucapsis. *Nova Hedwigia*, 441-456 (2016).

2 Krings, M. & Sergeev, V. N. A coccoid, colony-forming cyanobacterium from the Lower Devonian Rhynie chert that resembles Eucapsis (Synechococcales) and Entophysalis (Chroococcales). *Rev. Palaeobot. Palyn.* **268**, 65-71 (2019).

3 McKay, C. P. *et al.* Temperature and Moisture Conditions for Life in the Extreme Arid Region of the Atacama Desert: Four Years of Observations Including the El Niño of 1997–1998. *Astrobiology* **3**, 393-406 (2003).

4 Navarro-González, R. *et al.* Mars-like soils in the Atacama Desert, Chile, and the dry limit of microbial life. *Science* **302**, 1018-1021 (2003).

5 Pulschen, A. A. *et al.* UV-resistant yeasts isolated from a high-altitude volcanic area on the Atacama Desert as eukaryotic models for astrobiology. *Microbiology Open* **4**, 574-588 (2015).

6 Stevenson, A. *et al.* Multiplication of microbes below 0.690 water activity: implications for terrestrial and extraterrestrial life. *Environ. Microb.* **17**, 257-277 (2015).

7 Schulze-Makuch, D. *et al.* Transitory microbial habitat in the hyperarid Atacama Desert. *Proc. Nat. Acad. Sci.* **115**, 2670-2675 (2018).

8 Voigt, C., Klipsch, S., Herwartz, D., Chong, G. & Staubwasser, M. The spatial distribution of soluble salts in the surface soil of the Atacama Desert and their relationship to hyperaridity. *Global Planet. Change* **184**, 103077 (2020).

9 Singleton, P. *Bacteria in biology, biotechnology and medicine*. (John Wiley & Sons, 2004).

10 Shaw, M. K., Marr, A. G. & Ingraham, J. L. Determination of the Minimal Temperature for Growth of *Escherichia coli*. *J. Bacteriol.* **105**, 683-684 (1971).

11 Tamplin, M. L., Paoli, G., Marmer, B. S. & Phillips, J. Models of the behavior of Escherichia coli O157: H7 in raw sterile ground beef stored at 5 to 46 C. *Int. J. Food Microbio.* **100**, 335-344 (2005).

12 Van Derlinden, E., Bernaerts, K. & Van Impe, J. F. Dynamics of Escherichia coli at elevated temperatures: effect of temperature history and medium. *J. Appl. Microbio.* **104**, 438-453 (2008).

13 Philip, P. *et al.* Parallel substrate supply and pH stabilization for optimal screening of E. coli with the membrane-based fed-batch shake flask. *Microb. Cell Fact.* **17**, 69 (2018).

14 Stolper, D. A., Revsbech, N. P. & Canfield, D. E. Aerobic growth at nanomolar oxygen concentrations. *Proc. Nat. Acad. Sci.* **107**, 18755-18760 (2010).

15 Duc, M. T. L. *et al.* Isolation and Characterization of Bacteria Capable of Tolerating the Extreme Conditions of Clean Room Environments. *Appl Environ Microbiol* **73**, 2600-2611 (2007).

16 Schuerger, A., Richards, J., Newcombe, D. & Venkateswaran, K. Survival of seven Bacillus spp. under simulated Mars UV irradiation suggests minimum forward contamination around landing sites. *Int. J. Astrobiol. Suppl* **1**, 77 (2004).

17 Taylor, G. R. Space microbiology. *Annu Rev Microbiol* **28**, 121-137 (1974).

18 Venkateswaran, K. *et al.* Molecular microbial diversity of a spacecraft assembly facility. *Syst.Appl. Microb.* **24**, 311-320 (2001).

19 Barberán, A., Velazquez, H. C., Jones, S., Fierer, N. & Hallam, S. J. Hiding in Plain Sight: Mining Bacterial Species Records for Phenotypic Trait Information. *mSphere* **2**, e00237-00217 (2017).

20 Mykytczuk, N. C. *et al.* Bacterial growth at -15 degrees C; molecular insights from the permafrost bacterium Planococcus halocryophilus Or1. *Isme j* **7**, 1211-1226 (2013).

21 Mykytczuk, N. C. S., Wilhelm, R. C. & Whyte, L. G. Planococcus halocryophilus, an extreme sub-zero species from high Arctic permafrost. *Int. J. Syst. Evol. Microb.*, **62**, 1937-1944 (2012).

22 Heinz, J., Schirmack, J., Airo, A., Kounaves, S. P. & Schulze-Makuch, D. Enhanced Microbial Survivability in Subzero Brines. *Astrobiology* **18**, 1171-1180 (2018).

23 Stroble, S. T., McElhoney, K. M. & Kounaves, S. P. Comparison of the Phoenix Mars Lander WCL soil analyses with Antarctic Dry Valley soils, Mars meteorite EETA79001 sawdust, and a Mars simulant. *Icarus* **225**, 933-939 (2013).

24 Naz, N., Liu, D., Harandi, B. F. & Kounaves, S. P. Microbial Growth in Martian Soil Simulants Under Terrestrial Conditions: Guiding the Search for Life on Mars. *Astrobiology* **22**, 1210-1221 (2022).
